# Low Dopamine D2 Receptor Expression Drives Gene Networks Related to GABA, cAMP, Growth and Neuroinflammation in Striatal Indirect Pathway Neurons

**DOI:** 10.1016/j.bpsgos.2022.08.010

**Published:** 2022-09-08

**Authors:** Lucia Guerri, Lauren K. Dobbs, Daniel A. da Silva e Silva, Allen Meyers, Aaron Ge, Lea Lecaj, Caroline Djakuduel, Damien Islek, Dionisio Hipolito, Abdiel Badillo Martinez, Pei-Hong Shen, Cheryl A. Marietta, Susanna P. Garamszegi, Enrico Capobianco, Zhijie Jiang, Melanie Schwandt, Deborah C. Mash, Veronica A. Alvarez, David Goldman

**Affiliations:** a Laboratory of Neurogenetics, National Institute on Alcohol Abuse and Alcoholism (NIAAA), National Institutes of Health, Bethesda, Maryland; b Laboratory on Neurobiology of Compulsive Behaviors, NIAAA, National Institutes of Health, Bethesda, Maryland; c Department of Neuroscience, University of Texas at Austin, Austin, Texas; d Department of Neurology, University of Texas at Austin, Austin, Texas; e University of Maryland, College Park, Maryland; f Department of Neurology, Miller School of Medicine, University of Miami, Miami, Florida; g Institute for Data Science and Computing, University of Miami, Miami, Florida; h Office of the Clinical Director, NIAAA, National Institutes of Health, Bethesda, Maryland; i Department of Molecular and Cellular Pharmacology, Miller School of Medicine, University of Miami, Miami, Florida; j Intramural Research Program, National Institute on Drug Abuse, Baltimore, Maryland

**Keywords:** Addiction, Dopamine, Drd2, Indirect pathway, Psychiatric disorders, TRAP, Transcriptomics, Substance Use Disorder

## Abstract

**Background:**

A salient effect of addictive drugs is to hijack the dopamine reward system, an evolutionarily conserved driver of goal-directed behavior and learning. Reduced dopamine type 2 receptor availability in the striatum is an important pathophysiological mechanism for addiction that is both consequential and causal for other molecular, cellular, and neuronal network differences etiologic for this disorder. Here, we sought to identify gene expression changes attributable to innate low expression of the *Drd2* gene in the striatum and specific to striatal indirect medium spiny neurons (iMSNs).

**Methods:**

Cre-conditional, translating ribosome affinity purification (TRAP) was used to purify and analyze the translatome (ribosome-bound messenger RNA) of iMSNs from mice with low/heterozygous or wild-type *Drd2* expression in iMSNs. Complementary electrophysiological recordings and gene expression analysis of postmortem brain tissue from human cocaine users were performed.

**Results:**

Innate low expression of *Drd2* in iMSNs led to differential expression of genes involved in GABA (gamma-aminobutyric acid) and cAMP (cyclic adenosine monophosphate) signaling, neural growth, lipid metabolism, neural excitability, and inflammation. Creb1 was identified as a likely upstream regulator, among others. In human brain, expression of *FXYD2*, a modulatory subunit of the Na/K pump, was negatively correlated with *DRD2* messenger RNA expression. In iMSN-TRAP-*Drd2*HET mice, increased *Cartpt* and reduced *S100a10* (p11) expression recapitulated previous observations in cocaine paradigms. Electrophysiology experiments supported a higher GABA tone in iMSN-*Drd2*HET mice.

**Conclusions:**

This study provides strong molecular evidence that, in addiction, inhibition by the indirect pathway is constitutively enhanced through neural growth and increased GABA signaling.

Addictive drugs, particularly stimulants, are associated with increased dopamine release in the striatum (1, 2, 3). Humans with substance use disorders (SUDs) and animal models of addiction show blunted dopamine response (4) and maladaptive downregulation of dopamine D_2_ receptors (D2Rs) (5, 6, 7, 8). Furthermore, innate or induced low D2R confers vulnerability to addiction (9, 10, 11). However, interventions in animal models aimed at rescuing addictive phenotypes by restoring *Drd2* expression in the striatum have so far yielded mixed results (12, 13, 14, 15).

The heritability of SUDs ranges from 0.39 for hallucinogens to 0.72 for cocaine (16). Genome-wide association studies have implicated the dopamine receptor gene *DRD2* in alcohol and tobacco consumption (17,18) and other psychiatric disorders (19, 20, 21), although not driven by a frequently studied polymorphism (*DRD2*/ANKK1 rs1800497) (22).

D2Rs are mostly expressed on indirect pathway medium spiny neurons (iMSNs) that mediate behavioral inhibition (no-go signal). Conversely, dopamine D_1_ receptors (D1Rs) are mostly expressed on direct pathway MSNs (dMSNs) that mediate reward and the initiation of behavior (go signal) (23,24). In the context of addiction, sensitization and conditioned place preference to psychostimulants are generally elicited by dMSNs and inhibited by iMSNs (25, 26, 27, 28). Unbalanced striatal direct and indirect pathway signaling are hypothesized to be key to the pathophysiology of SUDs and other neuropsychiatric disorders such as schizophrenia and obsessive-compulsive disorder, and neurological conditions such as Parkinson’s and Huntington's disease. Therapies for several of these conditions target dopamine neurotransmission and alter signaling balance, partly restoring function but at times leading to troubling side effects (29, 30, 31). For example, Parkinson’s disease is treated with levodopa, a dopamine precursor, while dopamine D2R blockade is often used to treat both schizophrenia and Huntington’s disease. Despite its centrality to SUDs, there are currently no effective treatments that target dopamine signaling.

We previously reported that iMSNs partially or fully deficient for *Drd2* exhibit decreased dMSN excitability due to increased GABAergic collateral inhibition from iMSNs (32, 33, 34). This could contribute to addiction by increasing input needed for the striatum to select and maintain behavior, biasing the brain to pursue high-reward drug-associated cues and ignore lower intensity natural rewards.

In this study, we investigated gene expression changes in iMSNs that may explain the enhanced influence of the indirect pathway due to reduced *Drd2* expression. For this, we used a genetically modified mouse with innate low expression of *Drd2* selectively in iMSNs and analyzed its ribosome-bound transcriptome (translatome) with normal (2 functional alleles, iMSN-TRAP-*Drd2*WT) or heterozygous/low (1 functional allele, iMSN-TRAP-*Drd2*HET) expression of *Drd2*. iMSNs account for approximately 12% of all cells and approximately 47% of all neurons in the striatum, where the glia/neuron ratio is about 4:1 (35, 36, 37). Furthermore, iMSNs and dMSNs share most of their transcriptional signatures (38). Therefore, to reliably detect and quantify medium and low abundance transcripts specific to iMSNs, we used translating ribosome affinity purification (TRAP) (39) followed by a low-input RNA sequencing strategy and analysis.

## Methods and Materials

### Animals

Experimental protocols were approved by the National Institute on Alcohol Abuse and Alcoholism Animal Care and Use Committee. Animals were group-housed and kept in a standard light/dark cycle (6:00 AM on–8:00 PM off). Mice of both sexes were used and counterbalanced in all experiments. For RNA sequencing (RNAseq) and quantitative polymerase chain reaction (qPCR) validation experiments (*N*_total_ = 48 mice, age 2–5 months), Adora2a-Cre^+/−^ *Drd2*^loxP/wt^ (iMSN-*Drd2*HET) mice were crossed with Cre-conditional TRAP^+/+^ (JAX Stock No. 022367) to obtain Adora2a-Cre^+/−^ TRAP^+/−^ *Drd2*^loxP/wt^ and *Drd2*^wt/wt^ (iMSN-TRAP-*Drd2*HET and iMSN-TRAP-*Drd2*WT mice, respectively). Adora2a-Cre (40) (GENSAT, 036158-UCD) was used to target both the *Drd2* deletion (41) (*Drd2*^loxP^, JAX Stock No. 020631) and the TRAP system specifically to iMSNs. The *Drd2* gene was knocked out by a floxed deletion of exon 2, which contains the translation start codon. For electrophysiology experiments (*N* = 13 mice, age 4–7 months), both the Adora2a-Cre control and iMSN-*Drd2*HET mice were crossed with *Drd1a*-tdTomato (JAX Stock No. 016204).

### Translating Ribosome Affinity Purification

TRAP isolation of ribosome-bound messenger RNA (mRNA) from iMSNs in mice was performed as described (39) with the following differences: matrix per whole mouse striatum: 150 μL streptavidin MyOne T1 Dynabeads (No. 65601; Invitrogen) and 60 μL biotinylated protein L recombinant purified (No. 29997; Thermo Fisher Scientific). Briefly, whole mouse striata were collected (∼40 mg/sample), snap frozen into 1.5 mL tubes, and stored at −80 °C; homogenization was performed with 500 μL of tissue-lysis buffer, Fisherbrand RNase-Free Disposable Pellet Pestles individually wrapped (No. 12-141-364; Thermo Fisher Scientific) and prechilled, and a Pellet Pestle Cordless Motor (No. 749540-0000; Kimble). TRAP isolation was performed in 3 batches of 8 samples each (*n* = 24 samples; 1 sample = 1 mouse) for RNAseq and 2 batches of 12 samples each for independent qPCR validations (*n* = 24 samples). One sample from the RNAseq and 2 samples from the qPCR were excluded due to low quality. Total (ribosome-bound) RNA (totRNA) was purified from positive TRAP fractions (∼50–200 ng totRNA/sample) using PicoPure RNA Isolation Kit (No. KIT0204; Thermo Fisher Scientific), always including on-column DNase I treatment to remove genomic DNA contamination. The isolated iMSN mRNA from iMSN-TRAP-*Drd2*HET and iMSN-TRAP-*Drd2*WT mice is termed iMSN^D2HET^ and iMSN^D2WT^ here, respectively.

### Sequencing

The mRNA from 50 to 100 ng totRNA TRAP positive fractions was amplified and converted into double-stranded complementary DNA (cDNA) using Ovation RNA-seq System version 2 (No. 7102-32; NuGEN). Quantity and quality were assessed by Qubit (dsDNA HS Assay Kit, No. Q32854; Thermo Fisher Scientific) and Bioanalyzer (High Sensitivity DNA Kit, No. 5067-4626; Agilent Technologies), respectively. Approximately 200 ng double-stranded cDNA was sheared to approximately 200 bp fragments using Covaris microtubes (No. 520045) and sonicator (Covaris S2). Sequencing library and reagents were from IonTorrent by Thermo Fisher Scientific unless otherwise specified. Ion Plus Fragment Library Kit (No. 4471252) and Ion Xpress bar-code adapters 1–16 Kit (No. 4471250) were used to construct sequencing libraries. Quantity and quality of the sequencing libraries were assessed by Qubit, Bioanalyzer, and Ion Library TaqMan Quantitation Kit (No. 4468802). Ion P1 Hi-Q sequencing 200 Kit (No. A26433) and Ion P1 Hi-Q Template OT2 Kit (No. A26434) were used to template sequencing beads, and Ion P1 Chip Kit version 3 (No. A26771) was used for sequencing in an Ion Torrent Proton sequencer.

### Differentially Expressed Gene Analysis

**Figure 1:**
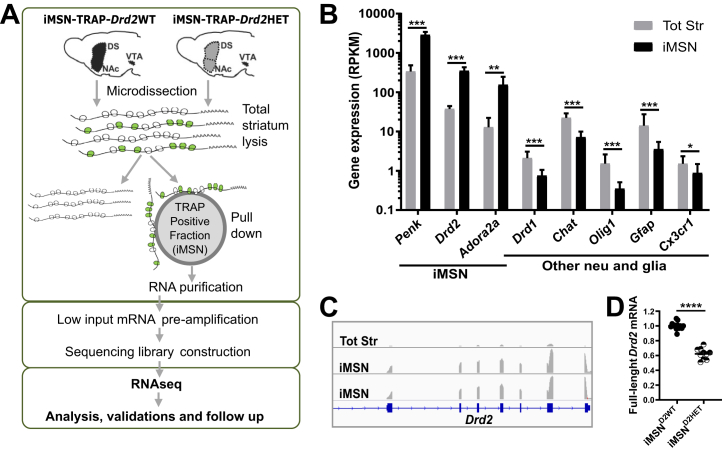
Enrichment of iMSN translatome signal in *Drd2*-low expression mice. **(A)** Experimental design. Whole striatum was microdissected from mice with WT or HET expression of *Drd2* and transgenic for a GFP-ribosomal subunit fusion protein conditionally expressed in *Drd2* neurons (iMSNs) for cell-specific mRNA purification. Conditional expression of the TRAP system and the floxed *Drd2* allele were driven by the *Adora2a* promoter. Isolation of actively translating mRNA (translatome) from iMSNs was achieved by pull down of GFP-ribosomes-associated mRNA followed by low input preamplification and sequencing. Differential gene expression from RNAseq samples was validated by qPCR (Figure S2 in Supplement 1) on an independent set of mice and samples. **(B)** Quantitative enrichment analysis of iMSN signal compared to total striatum by RNAseq shows a strong enrichment in iMSN translatome signal. **(C)***Drd2* exon signal coverage and enrichment in iMSN samples vs. total striatum, visualization on IGV (44). For comparison purposes, plots were set at the same scale. **(D)** Expression level of the full-length, functional *Drd2* mRNA calculated by mRNA exon coverage signal from the RNAseq data. GFP, green fluorescent protein; HET, heterozygous; IGV, Integrative Genomics Viewer; iMSN, indirect medium spiny neuron; mRNA, messenger RNA; NAc, nucleus accumbens; RNAseq, RNA sequencing; qPCR, quantitative polymerase chain reaction; RPKM, reads per kilobase of transcript per million mapped reads; Tot Str, total striatum; TRAP, translating ribosome affinity purification; VTA, ventral tegmental area; WT, wild-type.

An average of 27 million reads (±8 SD) were obtained per sample. Reads were mapped to mouse reference genome (mm10), and gene expression was modeled by generalized linear model using CLC Genomics Workbench (version 10; Qiagen Bioinformatics) in default settings. Protein coding genes (21,950 genes) were filtered by expression of reads per kilobase of exon per million mapped reads (RPKM) ≥ 2, resulting in 8332 genes considered expressed. A batch effect driven by the TRAP purification was corrected using ComBat (42) (Figure S1 in Supplement 1) from Bioconductor. EdgeR (43) was used for differential gene expression (DEG) analysis. The expression of functional *Drd2* mRNA in iMSN^D2HET^ (Figure 1D) was calculated by exon2/exon3-6 ratio, exons 3–6 being nondeleted in this construct and expressed normally. Nominal *p* values (i.e., unadjusted/uncorrected for multiple comparison) are denominated *p* and adjusted *p* values *p*_adj_. False discovery rate (= *p*_adj_) was used for DEG analysis.

### Gene Ontology Enrichment Analysis

The DEG analysis of the 8332 expressed genes was further analyzed with QIAGEN Ingenuity Pathway Analysis (IPA) (45). Given the lack of nonsense-mediated decay for the knocked-out allele of *Drd2* mRNA (exon-2 deletion), its expression value was divided in half before import to account for expression of the functional isoform. An IPA core analysis (based on expression and fold change [FC]) was performed using default settings except for the following: reference set = user dataset (8332 genes) and set cutoff = .05 *p-*value (unadjusted or nominal). Benjamini-Hochberg (= *p*_adj_) was used for enrichment analysis.

### Quantitative PCR

Quantification by qPCR of *Drd2* mRNA expression (Figure 1D) was calculated using cycle threshold (Ct) values of exon 2–3/exon 3–4 ratio and normalized to wild-type (WT) *Drd2* expression.

An independent set of 24 iMSNs-TRAP-*Drd2*WT and -HET mice was used for qPCR validation (Figure S2 in Supplement 1) of RNAseq gene expression results. qPCR samples were processed similarly to RNAseq samples until the double stranded cDNA was obtained. Then, 10 ng of double-stranded cDNA per well were used in Custom TaqMan Array Fast 96-well Plates (No. 4413261; Thermo Fisher Scientific), multiplexing with GAPDH-VIC and the gene target in FAM. TaqMan Fast Universal PCR Master Mix (No. 4352042; Thermo Fisher Scientific) and the QuantStudio 7 Flex Real-Time PCR System (Thermo Fisher Scientific) were used to run the fast 96-well plates. Two samples were excluded because of low quality. ComBat correction was used to address the qPCR TRAP-batch effect using ΔCt values.

### Gene Expression From Postmortem Brain Tissue From Subjects With Cocaine Abuse History

The gene expression levels for *DRD2* and *FXYD2* from an independent transcriptomics study (yet to be published) on postmortem human brain from the anterior caudate and the nucleus accumbens (NAc) from subjects with a severe cocaine abuse history (*n* = 25) and age-matched unaffected control subjects (*n* = 25) were obtained from the University of Miami Brain Endowment Bank. Details on samples and psychiatric pathology of these individuals have been reported elsewhere (46). Briefly, neuropathological specimens were obtained during routine autopsy. Total RNA was extracted from approximately 100 mg frozen caudate or ventral striatum, including on-column DNase I treatment. Approximately 500 ng totRNA/sample were sequenced at the Broad Institute (Cambridge, MA), using Illumina TruSeq library construction (Illumina, Inc.) including poly-A selection, run as 76-bp paired-end to a depth of approximately 50 million reads and aligned against Ensemble transcript reference.

### RNAscope

Sagittal brain slices (∼12 μm thick) of WT mice were used to detect *Drd1*, *Adora2a,* and *Fxyd2* gene expression in the striatum. RNAscope Multiplex Fluorescent Reagent version 2 (No. 323100; ACD) was used with dyes and dilutions as follows: 1:1500 *Drd1* (high expression gene)-Opal520, 1:1500 Adora2a (high expression gene)-Opal570 and 1:750 Fxyd2 (low expression gene)-Opal690 (Nos. FP1487001KT, FP1488001KT, and FP1497001KT, respectively; Akoya Bioscience), following HybEZ II Hybridization System (No. 321721) protocols.

### Electrophysiology

iMSN-*Drd2*HET mice or control mice (*Adora2a*-Cre) crossed with *Drd1a*-tdTomato mice (*n* = 13) were subjected to 5 consecutive days of cocaine (15 mg/kg, intraperitoneal injection) or saline treatment. Recordings were performed 2–5 days after the last injection. Whole-cell voltage-clamp recordings were measured from D1-MSNs, tdTomato positive neurons (*n* = 35, mean of 9 cells/3 mice) in the NAc core. Sagittal brain slices (240 μm) were prepared in ice-cold cutting solution and transferred to warm (31–33 °C) artificial cerebrospinal fluid bubbled with carbogen for 30 minutes. tdTomato+ dMSNs were held at −55 mV using glass electrodes (2.5–3.5 MΩ) filled with internal saline (pH ∼7.2, ∼300 mOsm). Data were acquired using Multiclamp 700B (Molecular Devices), filtered at 1 kHz and digitized at 5 kHz. All data were analyzed using pCLAMP (version 10.3; Clampfit). For solutions see Key Resources Table.

## Results

### *Drd2*-Low **i**MSN Translatome From a Mouse Model of Addiction

iMSN-specific, ribosome-bound mRNA (translatome) from a genetically diminished *Drd2* expression mouse model was isolated by TRAP, amplified, and sequenced (Figure 1A). Cre expression was driven by an *Adora2a* promoter in animals with one (HET) or no (WT) floxed alleles in exon 2 (containing the start codon) of the *Drd2* gene. The amplified and sequenced TRAP positive fractions showed strong enrichment for iMSN-specific transcripts (*Penk* 7.8-fold, *Drd2* 6.8-fold, *Adora2a* 2.6-fold) and reduced levels of non-iMSN transcripts compared to total striatum (Figure 1B, C; Figure S3 in Supplement 1). Expression of functional *Drd2* mRNA in iMSN^D2HET^ was approximately 60% of iMSN^D2WT^ (Figure 1D), while expression of *Drd2* exons other than exon 2 was unaltered, indicating lack of nonsense- mediated decay of *Drd2* mRNA transcribed from this construct.

### Differential Gene Expression in iMSN^D2HET^

**Figure 2:**
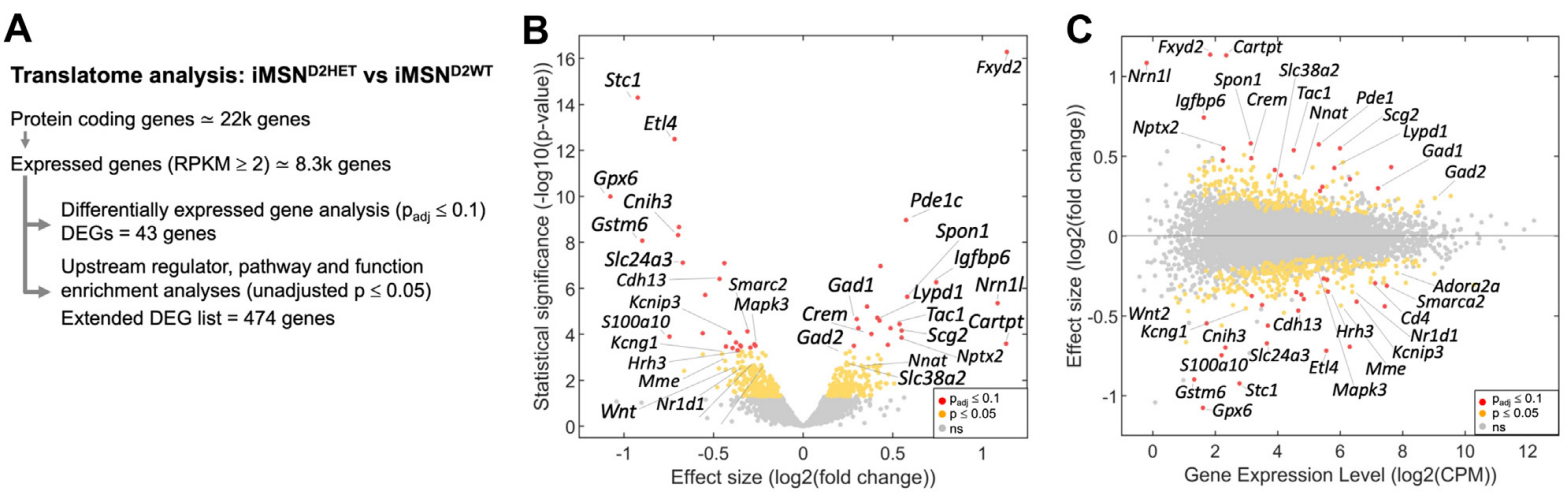
Differential gene expression in iMSN^D2HET^. **(A)** RNAseq bioinformatic analysis overview. **(B)** Volcano plot showing effect size (expression fold change) between iMSN^D2HET^ and iMSN^D2WT^ against statistical significance (red: genes with *p*_adj_ ≤ .1; yellow: *p* < .05) of all expressed genes in iMSN^D2HET^ (RPKM ≥ 2, ∼8300 genes). **(C)** MA plot showing effect size against mean level of expression (expressed in CPM) in WTs. CPM, counts per million; DEG, differentially expressed gene; iMSN, indirect medium spiny neuron; MA, minus average; ns, nonsignificant; RPKM, reads per kilobase of transcript per million mapped reads; WT, wild-type.

A total of 43 protein coding genes were differentially expressed in iMSN^D2HET^ compared with iMSN^D2WT^ (*p*_adj_ ≤ .1, *p* = 5 × 10^−4^), 19 being upregulated and 24 downregulated (Figure 2A–C and Table 1). To assess reproducibility, we used qPCR on TRAP-purified iMSN fractions from 24 iMSN-TRAP-*Drd2*HET and WT mice (independent from the 24 mice used for RNAseq). The 38 genes assessed for differential expression by both RNAseq and qPCR on independent samples were strongly correlated in both statistical significance (*r* = 0.55, *p* = 2.7 × 10^−4^) and FC values (*r* = 0.85, *p* = 10^−12^) (Figure S2 in Supplement 1), supporting the reliability of the RNAseq measurements.

**Table 1:** Differentially Expressed Genes in iMSN^D2HET^

Gene Symbol	FC	*p*_adj_	Gene Name and Notes	References
Upregulated				
*Fxyd2*	2.20	4 × 10^−13^	FXYD domain-containing ion transport regulator 2; modulatory γ-subunit of the Na/K pump; associated with schizophrenia and nicotine addiction	(47)
*Pde1c*	1.49	2 × 10^−6^	Phosphodiesterase 1C; degrades cAMP/cGMP; intronic variant associated with smoking phenotypes by GWAS	(48,49)
*Igfbp6*	1.67	3 × 10^−4^	Insulin-like growth factor binding protein 6; activates the MAPK signaling pathway; role in neurogenesis in the hippocampus	(50)
*Spon1*	1.50	.001	Spondin 1; secreted extracellular protein; role in neurite growth and axon guidance; genetic variant on its locus associated with cognitive decline by GWAS	(51,52)
*Nrn1l*	2.12	.002	Neuritin 1-like; extracellular protein; enhances neurite growth and neuronal survival	
*Gad1*	1.23	.010	Glutamate decarboxylase 1; key enzyme of GABA synthesis	
*Lypd1*	1.34	.010	Ly6/Plaur domain containing 1 *(*aka *Lynx2)*; modulator of nicotinic acetylcholine receptors; KO mouse model displays increased anxiety-related behavior	(53,54)
*Crem*	1.40	.020	cAMP-responsive element modulator; multiple alternative-splice isoforms acting as activators and repressors of transcription; paralog to *Creb1*; increased expression reduced impulsive action in an attention deficit hyperactivity disorder (ADHD) animal model	(55,56)
*Nptx2*	1.46	.039	Neuronal pentraxin 2; involved in synapse formation, specifically AMPA receptor clustering	(57)
*Cartpt*	2.19	.067	CART prepropeptide; modulates cocaine and dopamine effects in the striatum	(58,59)
Downregulated				
*Stc1*	−1.90	2 × 10^−11^	Stanniocalcin 1; glycoprotein involved in calcium/phosphate homeostasis	
*Gpx6*	−2.11	2 × 10^−7^	Glutathione peroxidase 6; neuroprotective against oxidative and other forms of stress; in a Huntington disease mouse model, upregulation in striatum improved behavioral and molecular phenotypes	(60,61)
*Fam163b*	−1.62	3 × 10^−6^	Family with sequence similarity 163, member B; membrane protein of unknown function; implicated in tobacco use disorder and stress	(62,63)
*Cnih3*	−1.62	6 × 10^−6^	Cornichon family AMPA receptor auxiliary protein 3	
*Gstm6*	−1.86	9 × 10^−6^	Glutathione S-transferase mu 6; neuroprotective against oxidative and other forms of stress	(60)
*Slc24a3*	−1.59	7 × 10^−5^	Solute carrier family 24, member 3; plasma-membrane potassium-dependent sodium-calcium exchanger, a druggable target for treatment; intronic variant associated with externalizing behavior by GWAS	(64,65)
*Id4*	−1.36	7 × 10^−5^	Inhibitor of DNA binding 4; inhibitory transcription factor; required for the correct timing of neural differentiation	(66)
*Cdh13*	−1.38	3 × 10^−4^	Cadherin 13 (aka *T*-cadherin); GPI-anchored cell adhesion molecule; inhibits neural growth and signals through ERK pathway; impacts GABAergic function; accumulative evidence of association with SUDs from GWAS	(65,67, 68, 69)
*S100a10*	−1.68	.036	S100 calcium binding protein A10 (calpactin); membrane protein involved in neurotransmitter transport; associated with depression and cocaine reward	(70)
*Mapk3*	−1.20	.071	Mitogen-activated protein kinase 3; MAPK/ERK pathway involved in growth	(32,57)

List of 20 genes upregulated (top) and downregulated (bottom) in iMSN^D2HET^ (*p*_adj_ ≤ 0.1) with relevant function notes; *p*_adj_ is the *p* value adjusted for multiple testing (false discovery rate).

ADHD, attention-deficit/hyperactivity disorder; cAMP, cyclic adenosine monophosphate; CART, cocaine- and amphetamine-regulated transcript protein; cGMP, cyclic guanosine monophosphate; ERK, extracellular signal-regulated kinase; FC, fold change; GABA, gamma-aminobutyric acid; GPI, glycosylphosphatidylinositol; GWAS, genome-wide association study; iMSN, indirect medium spiny neuron; KO, knockout; MAPK, mitogen-activated protein kinase; SUDs, substance use disorders.

Among the DEGs (*p*_adj_ ≤ .1), several have known pathway membership and/or functions, including cAMP (cyclic adenosine monophosphate) signaling (*Pde1c*, *Crem*, *Mapk3*, *Drd2*), calcium signaling (*Stc1*, *Slc24a3*, *Kcnip3*), cellular growth (*Id4*, *Cdh13*, *Igfbp6*, *Spon1*, *Nrn1l*, *Osbpl3*, *Crem*, *Ppm1l*, *Fgf11*), inflammation (*Stc1*, *Cdh13*, *Tac1*, *Scg2*, *S100a10*, *Hrh3*) and behavior (*Tac1*, *Crem*, *Sst*, *Hrh3*, *Mapk3*, *Adora2a*, *Mptx2*). See Table 1 for reported functions of 20 DEGs; see Figure S3 in Supplement 1 for striatal cell type expression; and see Table S1 in Supplement 2 for a complete gene list and values. *Gad1*, which encodes for a key GABA synthesis enzyme, was upregulated (FC = 1.23, *p*_adj_ = .01 and *p* = 2 × 10^−5^), and *Gad2* also but at trend level (FC = 1.18, *p*_adj_ = .12 and *p* = 7 × 10^−4^). *Cartpt* (FC = 2.19, *p*_ad_ = .07 and *p* = 3 × 10^−4^), associated with consumption of psychostimulants, was also up in iMSN^D2HET^.

### Gene Ontology Analysis

To identify complex biological functions affected by innate, low *Drd2* expression in iMSNs, we extended the list of genes of focus to include all those with suggestive or potential association (*p* ≤ .05, *n* = 474 genes). Of these 474 potentially differentially expressed genes, 214 were upregulated and 260 were downregulated (Figure 2) and will be referred to as the extended DEG list.

**Figure 3:**
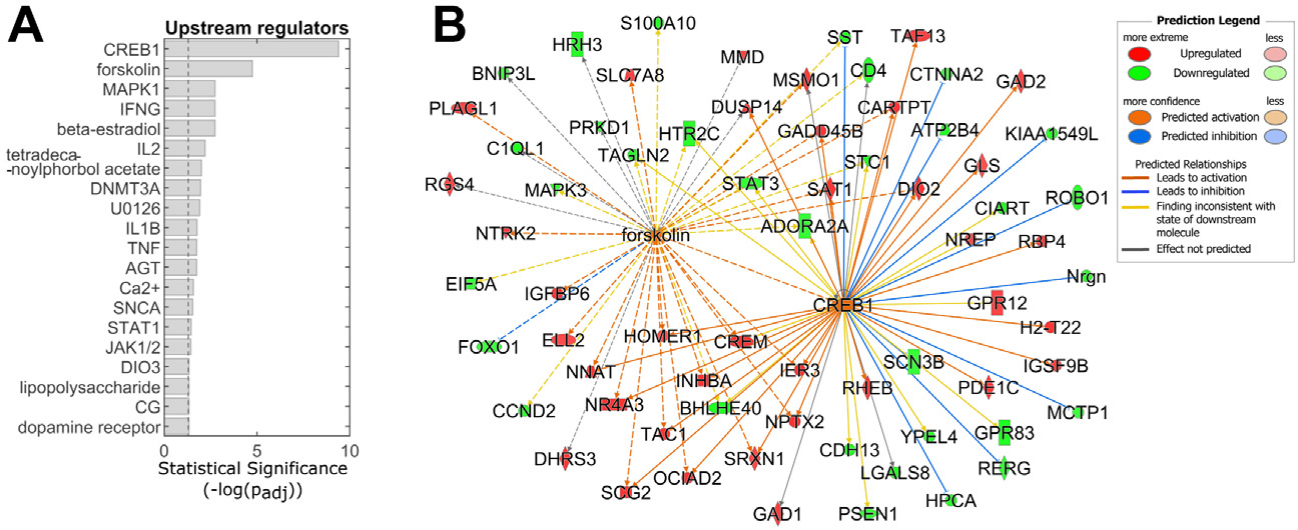
Creb1 and others were predicted upstream regulators of the observed expression profile in iMSN^D2HET^. **(A)** Upstream regulators predicted to have driven the expression changes observed in overlapping and nonoverlapping subsets of genes (see also Table S3 in Supplement 2) from the extended DEG list in iMSN^D2HET^. **(B)** Gene network showing Creb1 and forskolin (exogenous drug) targets within our dataset. DEG, differentially expressed gene; iMSN, indirect medium spiny neuron.

To identify molecules that could explain gene expression changes observed in iMSN^D2HET^, we performed upstream regulator analysis in IPA. Known Creb1 targets were strongly enriched (*p*_adj_ = 4 × 10^−10^, 52 out of 474 genes, i.e., 11%), and Creb1 function was strongly predicted to be increased (*z* = 2.6) (Figure 3; Table S3 in Supplement 2). Creb1 is a transcription factor well-recognized to be activated by cAMP/PKA signaling, facilitate synaptic plasticity, and play important roles in learning, long-term memory formation, and addiction (71, 72, 73). Other upstream regulators also predicted to be responsible for subsets of the observed differential expression pattern included beta-estradiol responsive to hormones, interferon gamma (IFNG), interleukin 2 (IL2) and interleukin 1β (IL1B), Ca^++^, and SNCA.

While the statistical significance for enrichment of canonical pathways did not survive correction for multiple testing (Table S4 in Supplement 2), glutamate dependent acid resistance (conversion of glutamate to GABA by Gad enzymes, *p* = .003) and cAMP-mediated signaling (*p* = .03) remain likely candidates to be affected in this low *Drd2* model. See Figure S4 in Supplement 1 for iMSN^D2HET^ DEGs in these signaling pathways.

**Figure 4:**
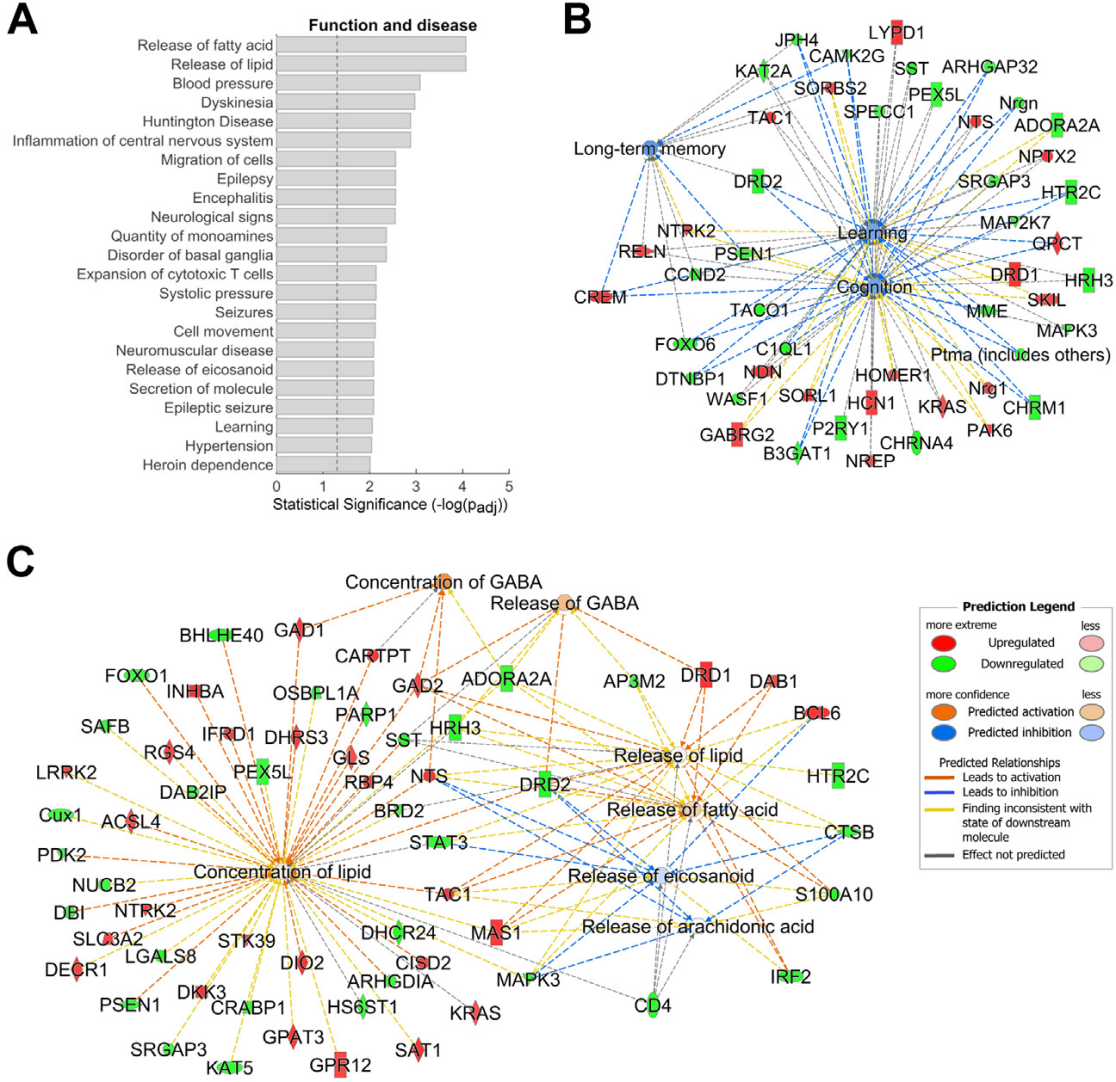
Disease and function enrichment analysis in iMSN^D2HET^. **(A)** Top disease and function annotations significantly enriched in the extended DEG list in iMSN^D2HET^. **(B)** Gene network for behavior-related functions: learning, cognition, and long-term memory. **(C)** Gene network for lipid metabolism-related functions: release of fatty acid, release of lipid, release of eicosanoid, concentration of GABA, release of GABA, and concentration of lipid. Functions in blue or orange indicate a predicted reduction or increase of function, respectively. For a complete list of genes, annotations, and values, see Table S5 in Supplement 2. DEG, differentially expressed gene; GABA, gamma-aminobutyric acid.

Further gene ontology analysis of the extended DEG list revealed strong enrichment in genes with diverse functions, including release of fatty acid and lipid and inflammation of the nervous system (Figure 4; Figures S5 in Suppement 1; Table S5 in Supplement 2). Dysregulated lipid metabolism in iMSNs has been associated with reward-related psychopathologies (74). Enriched disease and function terms with strong predicted activation (*z* score >2) include epilepsy or neurodevelopmental disorder, hypothermia, and secretion of catecholamine, while functions with strong predicted inhibition (*z* score < −2) include excitation of neurons, formation of neointima, and immune-mediated inflammatory disease.

### The Gene Network of *Drd2* in iMSN^D2HET^ Is Enriched in Cell-to-Cell Communication Molecules

To identify genes whose expression covaried with *Drd2* expression, we performed an independent weighted gene coexpression network analysis. In line with the IPA and further highlighting the impact of *Drd2* expression on the functional output of iMSNs, the module containing *Drd2* showed strong enrichment in genes involved in cell-to-cell communication, including ion transport, synaptic signaling and assembly, axon guidance, and matching molecular functions and cellular localizations (Figure S6 and Supplemental Methods in Supplement 1).

### *Fxyd2*, a Potentially Inhibitory Subunit of the Na/K Pump, Is Upregulated in iMSN^D2HET^

**Figure 5:**
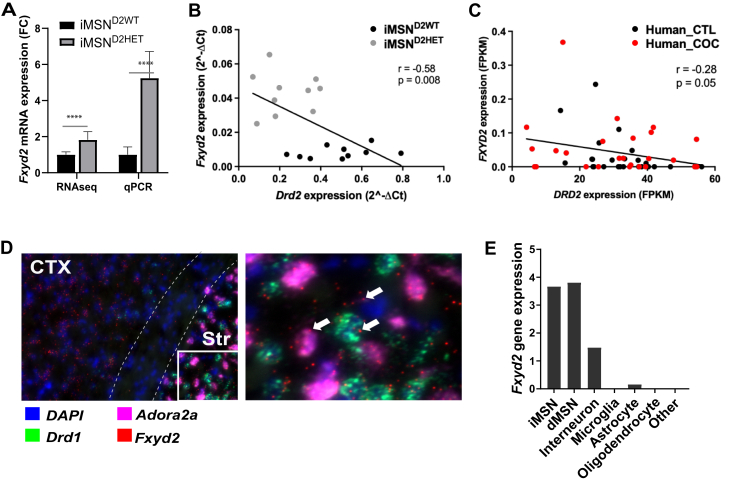
*FXYD2*, a potentially inhibitory subunit of the Na/K Pump, in the context of addiction in mouse and human. **(A)***Fxyd2* mRNA expression fold change in iMSN^D2HET^ relative to their WT counterparts in both RNAseq and qPCR independent samples (*p* = 5 × 10^-^^6^ and *p* < 1 × 10^-^^6^, respectively). **(B**-**C)** Correlated expression of *FXYD2* and *DRD2* mRNA in mouse and human striatum. **(B)** Mouse *Fxyd2* and *Drd2* mRNA expression correlation from qPCR on TRAP purified iMSN^D2HET^ and iMSN^D2WT^. **(C)** Human *FXYD2* and *DRD2* mRNA expression correlation from whole tissue RNAseq (FPKM) from postmortem caudate of individuals with severe cocaine abuse history (*n* = 25, red dots) and controls (*n* = 25, black dots). Pearson correlation values *r* and *p* are plotted. **(D)***Fxyd2* expression in mouse striatum and cortex by RNAscope suggests colocalization with *Drd1* (dMSNs) and *Adora2a* (iMSNs) expressing striatal neurons as well as unlabeled cells. **(E)** Cell-type expression of *Fxyd2* in mouse striatum from published single-cell transcriptome data (78) shows iMSN, dMSN, and interneuron expression. Values correspond to the mean expression. COC, cocaine; CTL, control; CTX, cortex; dMSNs, direct pathway MSNs; FC, fold change; FPKM, fragments per kilobase of transcript per million mapped reads; HET, heterozygous; iMSNs, indirect pathway MSNs; mRNA, messenger RNA; MSN, medium spiny neuron; qPCR, quantitative polymerase chain reaction; RNAseq, RNA sequencing; Str, striatum; TRAP, translating ribosome affinity purification; WT, wild-type.

*Fxyd2* was one of the genes with strongest differential expression (both in magnitude and statistical significance) (Figure 2B) in the iMSN^D2HET^ RNAseq and independent qPCR samples (Figure 5A). *Fxyd2* encodes the modulatory γ-subunit of the ATP-dependent Na/K pump that is essential for membrane potential and therefore neural excitability. *Fxyd2* is a phosphorylation target of PKA (75) and a member of the cAMP pathway (Kyoto Encyclopedia of Genes and Genome). In the brain, *Fxyd2* function is poorly understood but it modulates neuropathic pain through inhibition of the Na/K pump in nociceptive neurons (76,77).

We found that *FXYD2* and *DRD2* mRNA expression was inversely correlated in both mouse purified iMSNs (Figure 5B) and human postmortem caudate (whole-tissue RNAseq) of individuals with severe cocaine abuse history and control subjects (Figure 5C). This correlation was validated by independent cohorts from public repositories (Figure S7A in Supplement 1). *FXYD2* expression was not significantly different between cocaine and control groups (*p* = .11) (Figure S7B in Supplement 1); however, *FXYD2* expression in both mouse iMSNs (Figure 2C) and human is relatively low, and several human postmortem whole tissue samples had nondetectable levels (Figure 5C; Figure S7B); note axis expression scales for *DRD2* vs. *FXYD2*. RNAscope staining (Figure 5D) together with publicly available single-cell RNAseq data from mouse striatum (78) (Figure 5E) suggest *Fxyd2* expression in striatal iMSNs, dMSNs and to a lesser extent in interneurons, with little or no expression in glial populations.

Finally, no association approaching genome-wide significance was observed (Figure S7C in Supplement 1) between *FXYD2* genetic variations and lifetime overall alcohol use score in the National Institute on Alcohol Abuse and Alcoholism Clinical Center patient cohort (1181 cases and 546 controls). In publicly available knowledgebases such as Open Targets Genetics and GWAS Catalog, noncoding genetic variants near *FXYD2* were associated with human putamen volume (79, 80, 81).

### Heightened Striatal GABA Tone and Impaired Cocaine Response in *Drd2*-Low Mice

**Figure 6:**
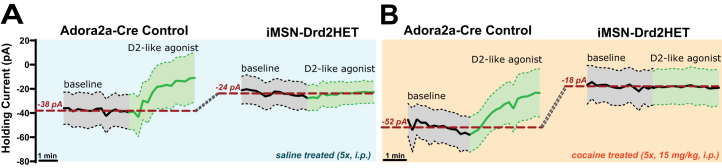
Functional impairment of D2R mediates electrophysiologic response in striatum of mice with low *Drd2* expression and with evidence for enhanced GABA tone. **(A)** Recordings from MSNs in control Adora2a-Cre mice show changes in holding current in response to application of the D2-like agonist quinpirole 1 μM (green trace). This response is severely impaired in mice with low *Drd2* expression (iMSNs-*Drd2*HET), which show no changes in holding current after quinpirole. Note that there are changes in baseline holding current in mice with low *Drd2* that are suggestive of enhanced GABAergic tone in iMSN-*Drd2*HET mice. **(B)** Cocaine pretreatment (5 days, 15 mg/kg) enhanced the average holding current in control mice from −38 to −52 pA but not in mice with low *Drd2* expression, which show persistently smaller holding currents than control mice consistent with increased GABAergic tone in mice with low D2Rs. D2R, dopamine type 2 receptor; GABA, gamma-aminobutyric acid; HET, heterozygous; iMSN, indirect medium spiny neuron.

dMSNs receive inhibitory GABA synapses from iMSN axon collaterals, which are suppressed by activation of D2Rs by the agonist quinpirole (32,82). Thus, electrophysiological recording of dMSNs provides a useful readout of GABA and D2R signaling in the striatum. We performed whole-cell voltage-clamp recordings from tdTomato+ dMSNs from the NAc core in sagittal brain slices of iMSN-*Drd2*HET and control mice. The D2R-like agonist quinpirole (Figure 6) (green trace) decreased the holding current in WT dMSNs (compared with baseline, gray trace) but exerted no effect in dMSNs from iMSN-*Drd*2HET mice. This loss of quinpirole-mediated response (in both saline- and cocaine-treated animals) provides further evidence of the functional impact of D2R reduction (83). Furthermore, the baseline average holding current of dMSNs in iMSN-*Drd2*HET was smaller than in control mice (−24/−18 pA vs. −38/−52 pA in saline and cocaine-treated groups, respectively), indicating more hyperpolarized dMSNs in iMSN-*Drd2*HET mice compared with WT, consistent with a heightened inhibitory GABA tone.

dMSNs in WT animals treated with repeated cocaine (15 mg/kg/day, 5 days) showed persistent depolarization at baseline compared with saline-treated control mice (−52 vs. −38 pA). More depolarized dMSN membrane potential after repeated cocaine suggests reduced GABA inhibition. This cocaine-induced depolarization was not seen in dMSNs of iMSN-*Drd2*HET mice. This suggests that D2Rs are required for mediating the effect of repeated cocaine in dMSNs, likely via suppression of striatal GABA release (32).

## Discussion

Reduced D2R availability in the striatum is both a consequence of and an etiologic contributor to SUD. Innate low D2R expression in young/adult mice appears to simultaneously stimulate the no-go pathway and inhibit the direct go pathway (34). Here, we sought to better understand the long-term consequences of innately reduced *Drd2* expression by analyzing the purified translatome (ribosome-bound transcriptome) of iMSNs from mice with WT or heterozygous expression of *Drd2* (selectively in iMSNs).

Consistent with our previous findings of increased striatal GABA signaling in mice with D2R deficiency (32,33), we found *Gad1* and *Gad2*, key genes encoding GABA-synthesis enzymes, upregulated and a predicted inhibition in stimulation of neurons function (*z* score = −2) by enrichment analysis in iMSN^D2HET^. Electrophysiological recordings showed that dMSNs were more inhibited at baseline in iMSN-*Drd2*HET mice compared with WT control mice.

Possibly related to the increased GABA tone, *Fxyd2*, a potentially inhibitory subunit of the Na/K pump, was robustly upregulated in iMSN^D2HET^. Its modulatory role is cell-type and environment dependent (84) but was shown to be inhibitory in nociceptive neurons (76,77) where loss of *Fxyd2* resulted in neural hyperpolarization (77). If *Fxyd2* also inhibits the Na/K pump in iMSNs, its upregulation could result in increased iMSN excitability. This could be an adaptive response by iMSNs to counteract the heightened GABA tone.

An evolutionarily conserved negative correlation between *FXYD2* and *DRD2* mRNA expression was observed in both our mouse model and in human postmortem caudate with and without severe cocaine use history. This correlation could be explained by a genetic variant at the *DRD2* locus affecting *FXYD2* expression (i.e., expression quantitative trait locus), which we could not test due to the limited detectability of *FXYD2* in the brain.

iMSN^D2HET^ displayed differential expression of genes associated with cAMP signaling and cellular growth. *Mapk3* (Erk) modulates processes that overlap with those of cAMP-dependent *Pka*. We have previously observed a shift from Pka toward the Erk/Mapk signaling pathway in dMSNs from iMSN-*Drd2*KO mice (83). Here, iMSN^D2HET^ showed downregulation of *Mapk3* (Erk), suggesting an opposing shift in iMSNs.

Creb1, a transcription factor well-known for its role in growth, learning, and synaptic reinforcement, was predicted to be an upstream regulator (*p*_adj_ = 4 × 10^−10^) with increased activity (*z* score = 2.6). There was no differential expression of the *Creb1* gene at the time of sample collection, suggesting it exerted its effect at an earlier time point. Studies across development are needed to test the hypothesis of differential neural growth of iMSNs over dMSNs in iMSN-*Drd2*HET mice.

Several genes were associated with inflammation. Inflammation of the nervous system was among the most significantly enriched functions, and immune-mediated inflammatory disease was predicted to be inhibited (Figure 4; Table S5 in Supplement 2). There is a growing body of evidence for a role of neuroinflammation (not accompanied by infiltration of peripheral immune cells) in several psychiatric disorders, including SUDs (85, 86, 87). Transcription profiling of rhesus macaques following long-term (∼100 days) cocaine self-administration revealed upregulation of neuroinflammation-related genes in the NAc but not in the ventral tegmental area (88), showing differential neuroinflammation response to drugs of abuse across brain regions. Anti-inflammatory strategies to treat SUDs show promising results including improvements in behavioral and cognitive outcomes (89, 90, 91) but with negative as well as positive outcomes in early clinical trials (92).

iMSN^D2HET^ also displayed upregulated *Cartpt* and downregulated *S100a10*, reproducing gene expression changes observed in mouse studies in cocaine paradigms. In the striatum, cocaine and amphetamine upregulate *Cartpt* mRNA expression and CART peptides, and co-administration of CART peptide and cocaine into the NAc reduced cocaine-induced locomotor activation (58, 93, 94), suggesting a compensatory role for *Cartpt*. Conversely, mice with reduced *S100a10* (p11), a small calcium-binding protein involved in neurotransmitter transport, have an enhanced cocaine conditioned place preference, while p11 overexpression in the NAc reduced it (70). Mice with low p11 also exhibited depression-like behavior, and its restoration in the NAc recovered the phenotype (95).

Recent studies show gene expression biases of *Drd2* expressing neurons across regions of the striatum (96) and compared to *Drd1* neurons (97). Among our DEGs, *Gstm6*, *Fam163b*, *Etl4*, *Spoon1* (96), and *S100a10* (96,97) were preferentially expressed in *Drd2* neurons from the dorsal striatum, while *Lypd1*, a modulator of nicotinic acetylcholine receptors, showed increased expression in the NAc (96,97) and in *Drd1* neurons (97). Further studies are needed to translate these observations into function.

An intrinsic challenge of this study was the observed and expected small-magnitude changes between groups because they only partially differ in the expression of a modulatory signaling receptor (*Drd2*). Gene expression changes translate into function whether they are big or small in magnitude, but small-magnitude differences increase the number of samples needed to statistically resolve them. Regarding technical strengths, low-input sequencing of TRAP positive fractions allowed us to study the iMSNs (∼12% of cells in the striatum) translatome and reliably detect transcripts with medium and low levels of expression that would have been lost via whole-tissue RNAseq. This was critical to our research, particularly given the functionally opposing roles of dMSNs and iMSNs, which also share a highly overlapping molecular profile.

In summary, we identified numerous differentially expressed genes in iMSNs driven by low D2R expression, modeling an observed trait of addiction in humans. We provided molecular evidence for enhanced GABA transmission. We identified enrichment in lipid metabolism, growth-related genes, cell-to-cell communication, and synaptic components that may reflect neural growth and/or increased maintenance given previous active growth. Striatal development in iMSN^D2HET^ and by extension in people with genetically encoded lower levels of D2R expression may feature an increased number of inhibitory iMSN-dMSN axon collaterals and an increased number of GABAergic presynaptic vesicles and release. Thus, our results further support an addiction model in which low D2R expression drives changes in the striatal microcircuitry and help explain its contribution to an enhanced indirect pathway and SUD-related behaviors.

## Supplementary Material

Supplementary Tables

Key Resources Table
