# Supplementary material for: Low Dopamine D2 Receptor Expression Drives Gene Networks Related to GABA, cAMP, Growth and Neuroinflammation in Striatal Indirect Pathway Neurons
Source: Biol Psychiatry Glob Open Sci. 2022 Sep 8;3(4):1104–15. doi: 10.1016/j.bpsgos.2022.08.010 (PMC10593893; doi:10.1016/j.bpsgos.2022.08.010)
Supplement: Supplement [file mmc1.pdf]

SUPPLEMENTARY INFORMATION

Low Dopamine D_2 Receptor Expression Drives Gene Networks Related to GABA, Cyclic AMP, Growth and Neuroinflammation in Striatal Indirect Pathway Neurons

SUPPLEMENTARY FIGURES

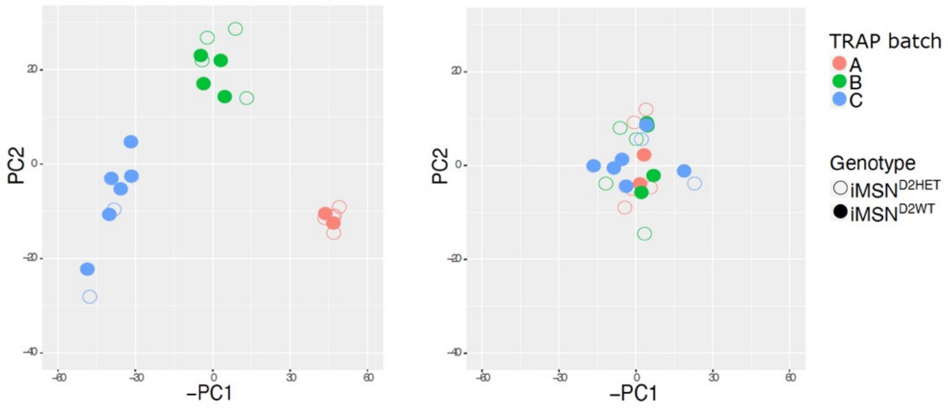

Sup Fig 1. ComBat correction of the TRAP purification-related batch effect. Principal Component Analysis (PCA) on RNAseq samples before (left) and after (right) ComBat normalization for batch correction. PCA before normalization shows clustering associated with the TRAP purification batch.

A

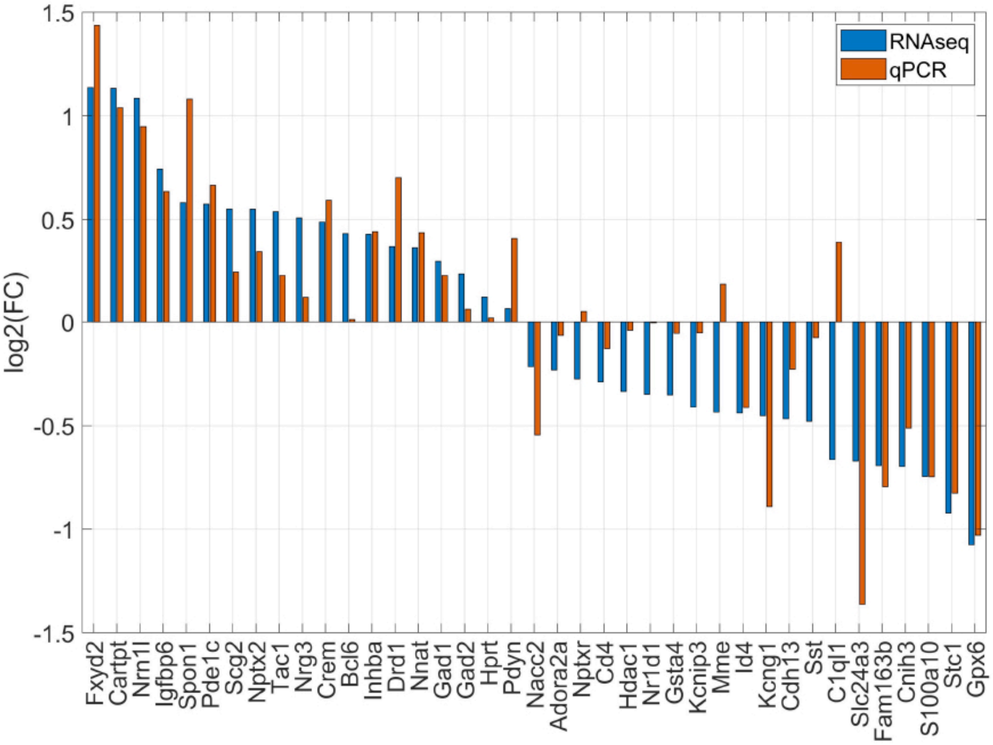

B

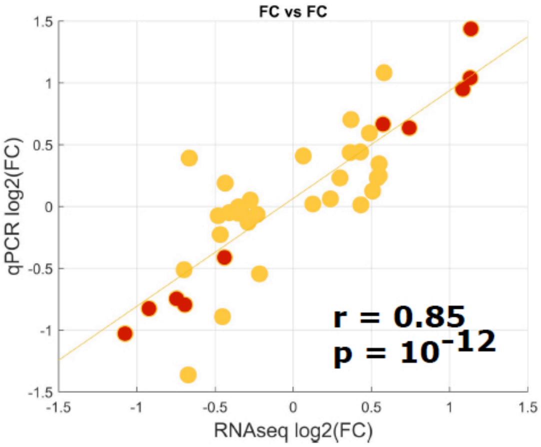

Sup Fig 2. qPCR validation of differential gene expression in $iMSN^{D2HET}$ and $iMSN^{D2WT}$ samples from independent mice. A) Fold change (FC) means are plotted for RNAseq and qPCR. B) Correlation between gene FC (magnitude and directionality) in RNAseq and qPCR. Red dots correspond to DEGs ($p_{adj} \leq 0.1$) and orange dots to genes with potential statistical association ($p \leq 0.05$).

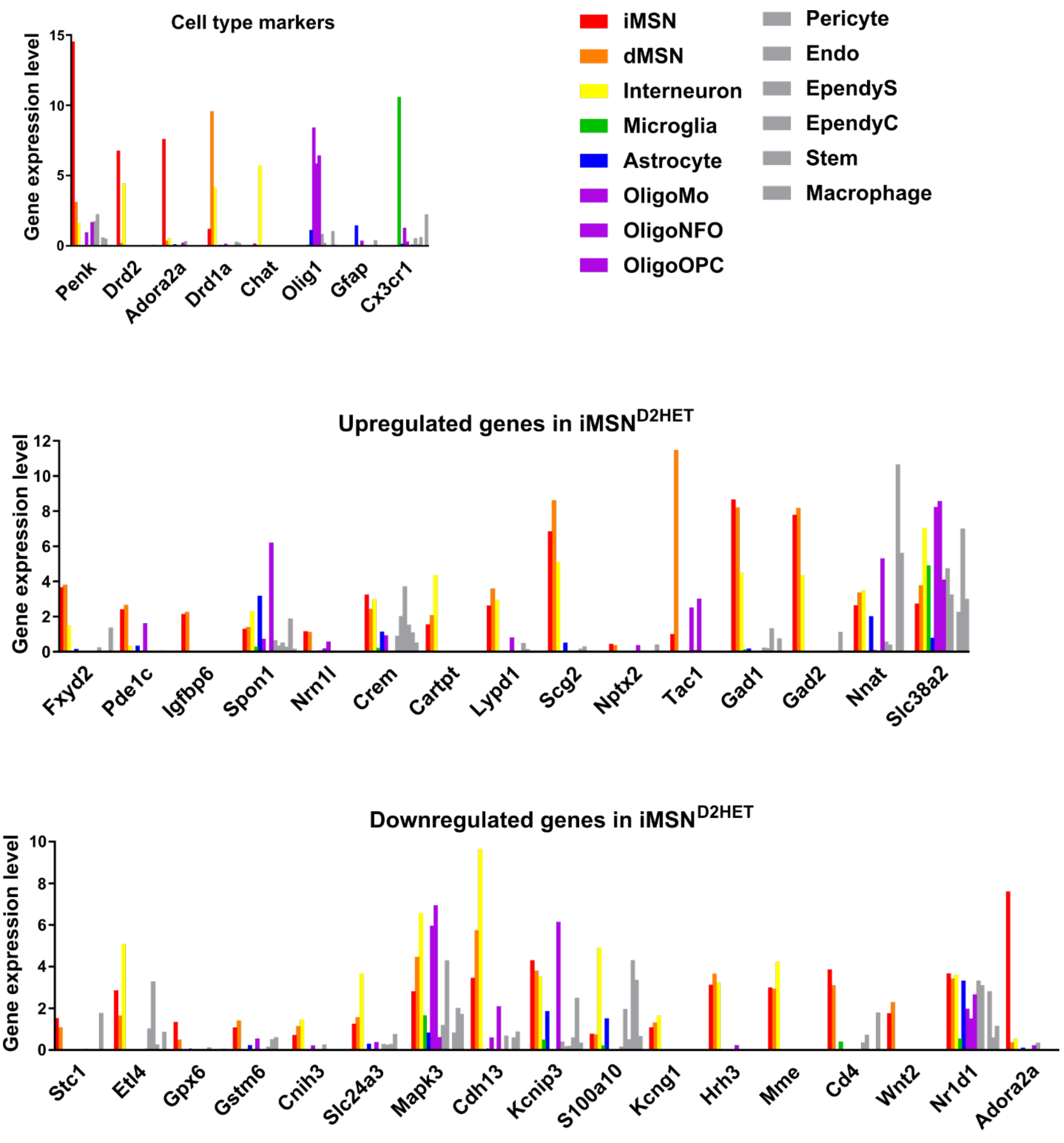

Sup Fig 3. Cell type expression profile in mouse striatum of genes of interest. Mean of expression values (RPKM) from published data of single cell RNAseq in mouse striatum. Cell type expression profile (top plot) of known cell type markers used in figure 1B, and upregulated (middle plot) and downregulated (bottom plot) genes in $iMSN^{D2HET}$.

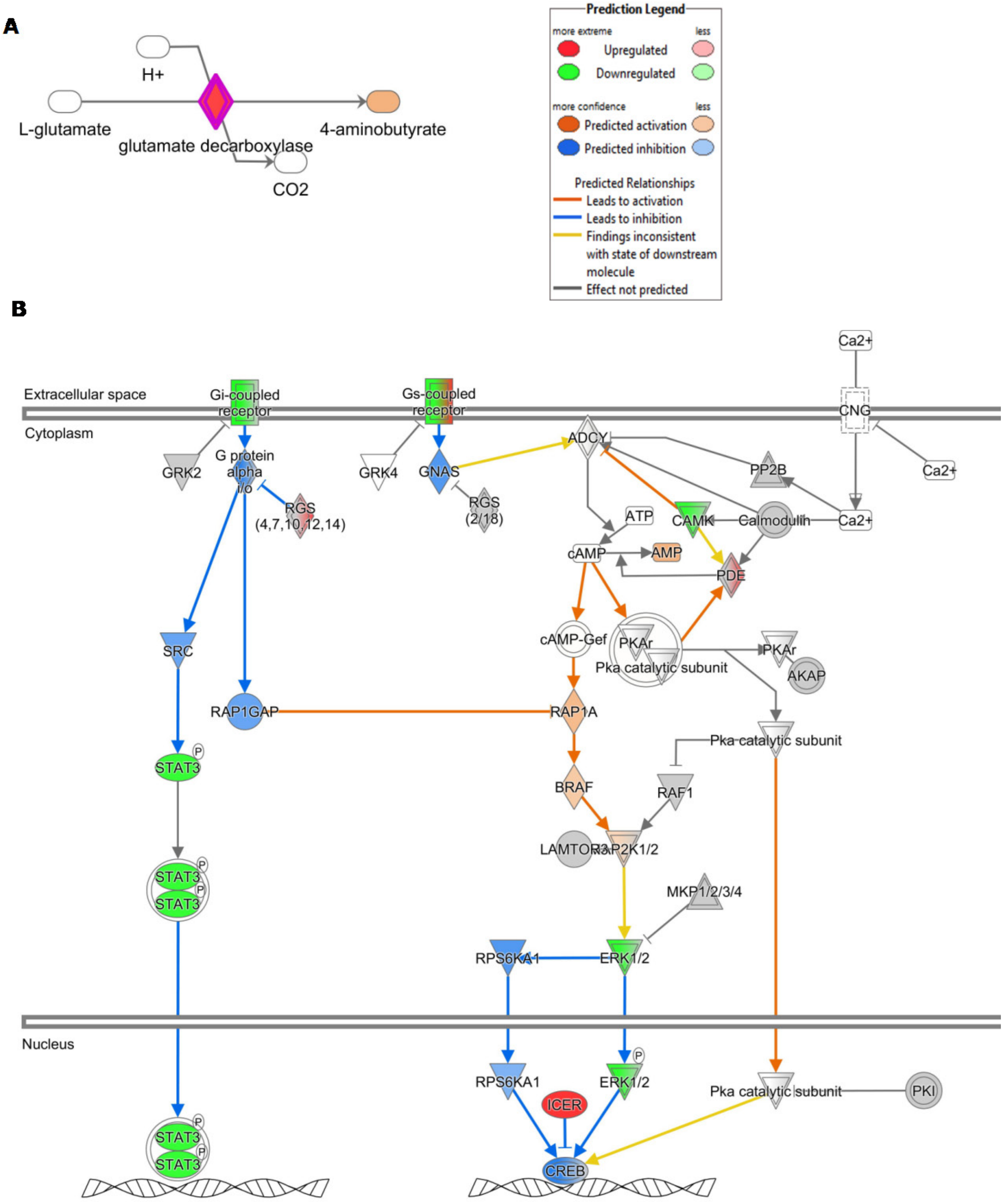

Sup Fig 4. Canonical pathway enrichment analysis. A) GABA synthesis pathway (IPA "Glutamate Dependent Acid Resistance") by *Gad1* and *Gad2* enzyme coding genes (in red). B) cAMP-signaling pathway.

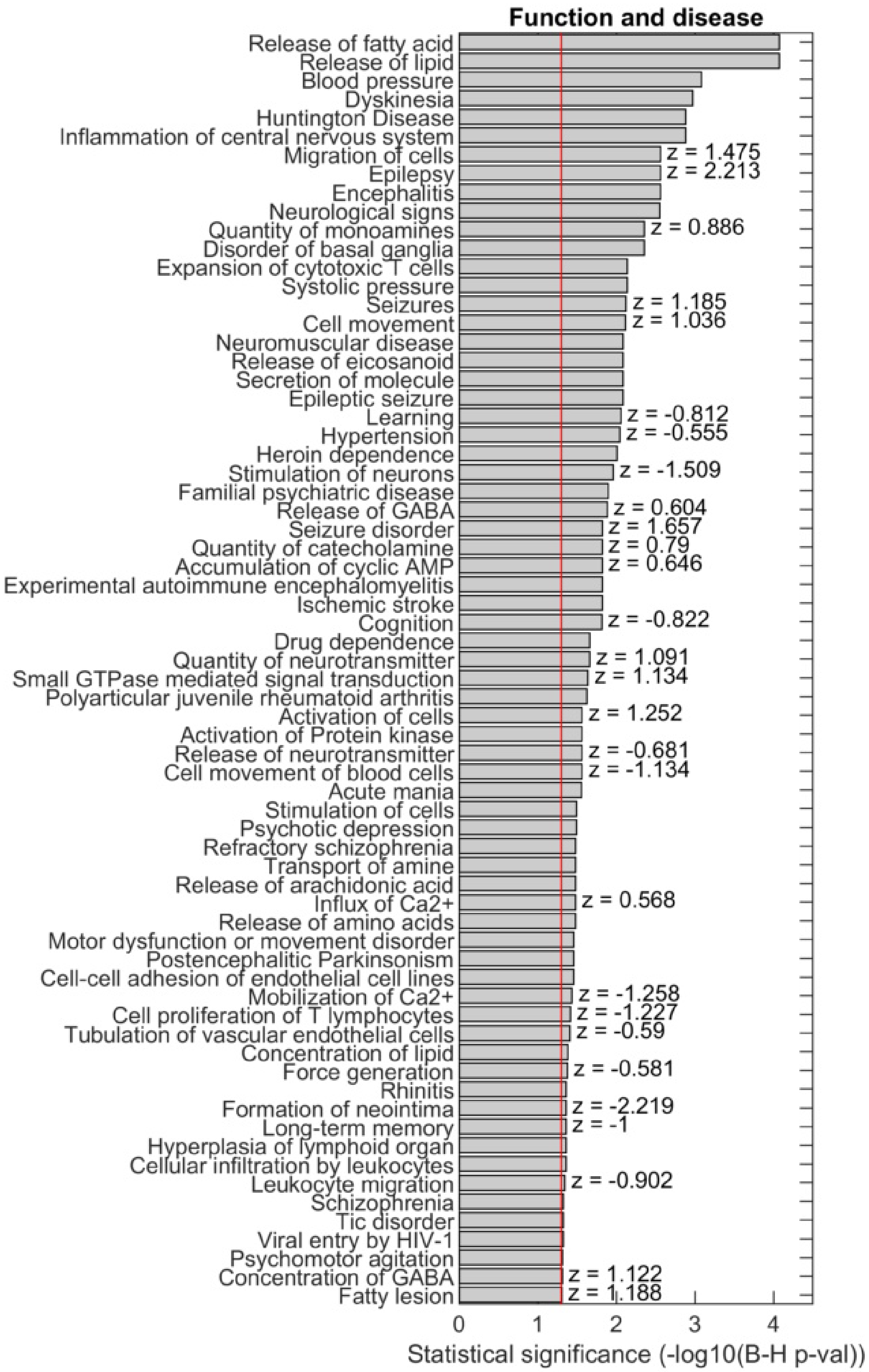

Sup Fig 5. Function and disease enrichment analysis in iMSN^{D2HET}. Enriched "Disease and Function" annotations by IPA, with respective z-scores that indicate the predicted directionality of the change. IPA considerers an absolute value of 2 for statistical significance (-2 ≥ z-score ≥ 2). The extended DEG list (p ≤ 0.05, 474 genes) was used. Red line indicates B-H p_{adj} = 0.05.

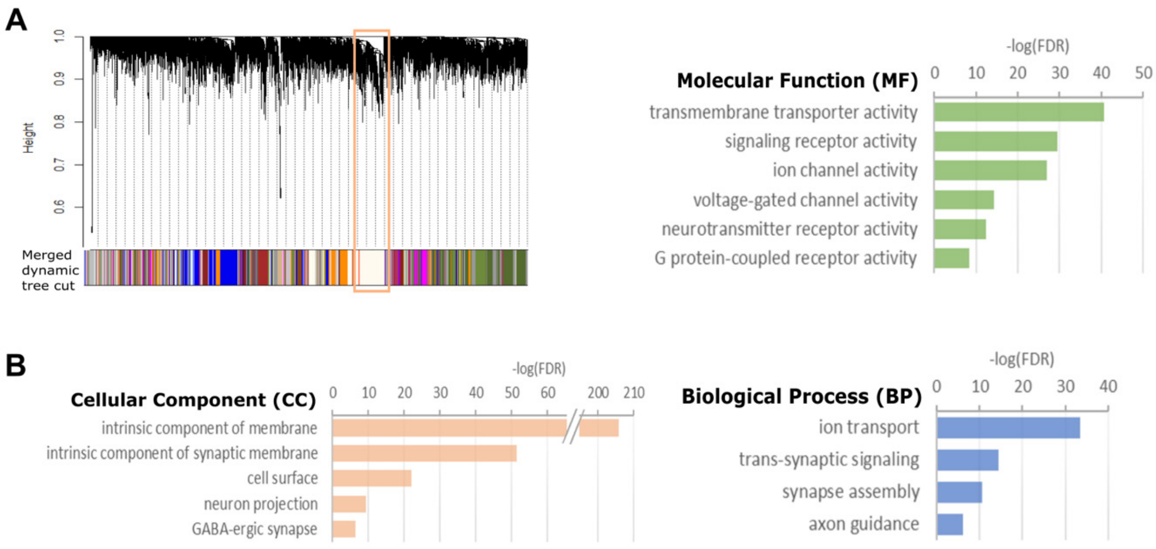

Sup Fig 6. Weighted Gene Co-expression Network Analysis (WGCNA). A) Co-expression dendrogram and gene modules, including all expressed genes in our dataset (~8.3k genes) and across all iMSN samples from iMSN-TRAP-*Drd2*HET and -*Drd2*WT mice. Highlighted with an orange box is the module or cluster containing *Drd2* (“floralwhite”). B) Gene Ontology (GO) term enrichment of the genes in the “floralwhite” module including *Drd2*.

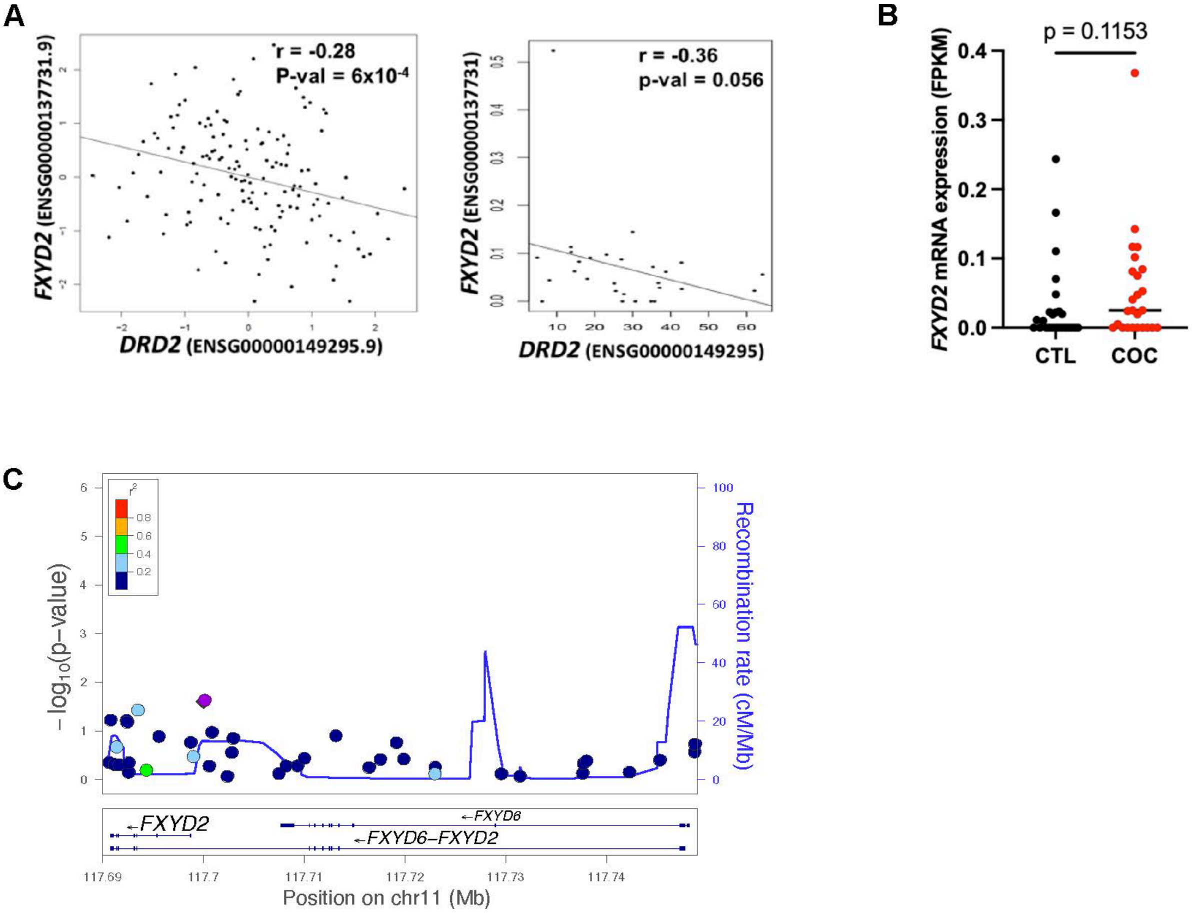

Sup Fig 7. *FXYD2*, a modulatory subunit of the Na/K Pump, in the context of addiction in mouse and human. A) Validation cohorts for *FXYD2* and *DRD2* mRNA expression correlation from RNAseq publicly available datasets: GTEx (left plot, caudate, n = 144, normalized expression) and BrainSpan (right plot, striatum, including embryonic development to adulthood, n = 28, RPKM). B) Human *FXYD2* mRNA expression from whole tissue RNAseq (FPKM) from postmortem caudate of individuals with severe cocaine abuse history (n=25, red dots) and controls (n=25, black dots). C) Gene association study in patients with Alcohol Use Disorders (AUD) shows no significant association at genome wide level between *FXYD2* genotypes and overall score for lifetime AUD. A lifetime overall AUD score based on the Structured Clinical Interview for the DSM (SCID) questionnaire was generated for each patient and control and run against their *FXYD2* gene variants.

SUPPLEMENTARY METHODS

Weighted Gene Co-Expression Network Analysis (WGCNA)

The WGCNA R package (1) was used to conduct co-expression gene network analysis. For this all genes above the expression cutoff in iMSNs (~8.3k genes) were included, and all samples ($iMSN^{D2HET}$ and $iMSN^{D2WT}$) were analyzed together regardless of genotype (Sup Fig 6). Given the hypothesis that genes whose expression levels co-vary share biological functions, we investigated the module that contained *Drd2* (labeled "floralwhite").

Commands in the WGCNA package were run on default settings unless otherwise specified. A soft-threshold power of 14 was used to satisfy approximation to scale-free topology ($R^2 \approx 0.907$). Given that we used RNAseq data from a purified cell-type, an unsigned network was generated to allow up and down-regulated genes to cluster together. The adjacency matrix was obtained using biweighted midcorrelation. The topological overlap matrix was then derived from the adjacency matrix using the TOMSimilarity function. The network dendrogram was constructed through the average linkage hierarchical clustering of the dissimilarity topological overlap matrix derived from the TOM (1-TOM). Modules were defined with the cutreeDynamic function, with deepsplit set to 2, pamStage set to true, and a minimum module size of 20 genes. Modules with eigengene correlations of 0.75 or higher were then merged together using the mergeCloseModules function. Gene Ontology (GO) enrichment analysis was performed using the enrichmentAnalysis function with a Bonferroni threshold of 1×10^{-4} from the anRichment package.

Replication cohorts for *DRD2* and *FXYD2* expression from postmortem human brain from public repositories

Two replication cohorts for *DRD2* and *FXYD2* mRNA expression correlation from postmortem human brain were obtained from public repositories (Sup Fig 7A). Normalized expression values from adult caudate were obtained from GTEx (N = 144), and from striatum across developmental timepoints from Brain Span (N = 27).

Gene Association Study: *FXYD2*

A conventional case/control association analysis was performed between *FXYD2* genetic variants and a lifetime score for Alcohol Use Disorder (AUD) on the European population from the NIAAA Clinical Center patient cohort (1181 cases, 546 control). The lifetime AUD score was calculated based on the Structured Clinical Interview (SCID) questionnaire for the Diagnostic and Statistical Manual of Mental Disorders (DSM–5). The analysis was performed by logistic regression model, and Ancestry Informative Marker (AIM) scores were included as covariates. 41 genotyped single nucleotide polymorphisms on the

FXYD2 locus (from Illumina Bead Arrays) were analyzed. The plot was generated with LocusZoom.

SUPPLEMENTARY REFERENCE

1. Langfelder P, Horvath S (2008): WGCNA: an R package for weighted correlation network analysis. *BMC Bioinformatics* 9: 559.
